# Poly(ethylene glycol)-Lipid-Conjugated Antibodies Enhance Dendritic Cell Phagocytosis of Apoptotic Cancer Cells

**DOI:** 10.3390/ph5050405

**Published:** 2012-04-26

**Authors:** Urara Tomita, Satoshi Yamaguchi, Yoichiro Sugimoto, Satoshi Takamori, Teruyuki Nagamune

**Affiliations:** 1 Department of Chemistry and Biotechnology, School of Engineering, The University of Tokyo, 7-3-1 Hongo, Bunkyo-ku, Tokyo 113-8656, Japan; 2 Department of Bioengineering, School of Engineering, The University of Tokyo, 7-3-1 Hongo, Bunkyo-ku, Tokyo 113-8656, Japan

**Keywords:** antibody conjugates, dendritic cell vaccine, antibody-dependent cell phagocytosis, poly(ethylene glycol)-lipid

## Abstract

A simple method for attaching immunoglobulin G (IgG) on the cell surface was successfully developed for enhancing phagocytosis of apoptotic tumor cells (ATCs) by dendritic cells (DCs) *ex vivo*. By conjugating with a poly(ethylene glycol) (PEG)-lipid, named the biocompatible anchor for the membrane (BAM), arbitrary IgG could be incorporated into the cell membrane. In particular, when IgG-BAM conjugates were prepared at the optimal molar ratio of IgG to BAM (1 to 20), almost all cells were efficiently modified with IgG by treatment with IgG-BAM. This simple method was successfully applied to four types of mammalian cells. Furthermore, treatment of ATCs with the IgG-BAM conjugate increased the phagocytosis ratio of ATCs by DCs two-fold when compared to no treatment. This phagocytosis-enhancing effect was nearly identical to treatment with a tumor-specific IgG. Thus, without employing the tumor-specific IgG, which is difficult to obtain for any tumor cells and is expensive, the present method could opsonize ATC with the use of arbitrary IgG. The results strongly indicate that IgG-BAM treatment represents a promising method for opsonizing ATC with human serum IgG, and that this approach will lead to objective clinical responses in DC vaccines.

## 1. Introduction

The dendritic cell (DC) vaccine has become a promising strategy in adoptive cell therapy, particularly in cancer immunotherapy [1,2]. For DC vaccines against cancer, DCs are loaded with tumor antigens *ex vivo*, and upon administration into patients, the DCs with presenting antigenic epitopes can activate anti-tumor effector T and B lymphocytes, and are capable of promoting natural killer (NK) T cells or NK cell activation [3]. Subsequently, these activated mediator and effector cells are reported to reject tumors in numerous studies using animal models [4,5]. However, although a large number of patients had received DC vaccines in clinical trials, only a limited number of objective clinical responses have been reported [1,4,5]. This disappointing result has prompted further studies examining each step in DC vaccination, such as source and *ex vivo* manipulation of DCs, antigen (Ag)-loading of DCs, timing and dosing of DC administration and the route of DC administration. In particular, the Ag-loading of DCs is critical because the choice of Ag influences the specificity of the immune response, and the choice of adjuvant defines the quality and magnitude of the anti-tumor response [4,6].

The ideal tumor Ag for a DC vaccine should induce a broad repertoire of anti-tumor immunocytes with high avidity for tumor cells. However, in current trials, single MHC-class-I-restricted synthetic peptides are the most commonly used therapeutic Ags. The use of such single synthetic peptides is restrictive because it provides a limited repertoire, such as the possible occurrence of promoting tumor antigen escape variants and the limited activation of cytotoxic T-lymphocytes (CTL) [1,7]. In addition, synthetic peptides-based DC vaccinations require prior knowledge of the sequence of the suitable-antigenic epitopes [6]. Conversely, over a decade ago, whole apoptotic tumor cells (ATCs) were found to represent promising Ag sources, because ATCs contain a broad spectrum of known and unknown Ags [6,8,9]. The use of ATCs potentially overcomes the limitations of synthetic peptides-based DC vaccinations. Such an approach has been presented, in which, DCs loaded with ATCs could induce activation of CTLs and helper T cells, as well as NK and γδ T cells [6]. Previous reports have suggested that activation of NK cells by DCs may be required for antitumor immunity in MHC-class-I-negative malignant tumors [10], and under certain conditions for successful CTL activation [11]. Thus, ATCs may provide a useful and effective source of antigens for overcoming the current problems associated with DC vaccine strategies.

Ag-immunoglobulin G (IgG) complexes can efficiently sensitize immature DCs for activation of both CTLs and helper T cells in DC vaccine-based antitumor immunity [12,13]. IgG-complexed Ags are internalized into DCs through the uptake mediated by receptors for the Fc domain of IgG (FcγR), and then, the antigenic peptides derived from the Ags are presented on MHC class I as well as MHC class II cells. In addition, the use of IgG can promote Ag presentation 100-fold more efficiently over pinocytosis of soluble Ags and effectively induce DC maturation [12]. These advantages of Ag-IgG complexes were confirmed in a ATCs-based DC vaccination. Akiyama *et al.* reported that the ATCs bearing IgG, which were prepared by chemically modifying cell membranes with IgG, provided a more efficient vaccination against tumors [7]. However, chemical modification of the membrane of ATCs may alter the properties of antigens derived from tumor membrane proteins. Instead of chemical modification of IgG, tumor-specific antibodies can be attached onto the surface of tumor cells via their molecular recognition. However, the employment of antigen-specific antibodies is expensive and therefore increases the medical cost [13,14]. Consequently, alternative methods of displaying IgG on the ATC surface is likely to be required for the development of inexpensive and effective DC vaccines.

**Figure 1:**
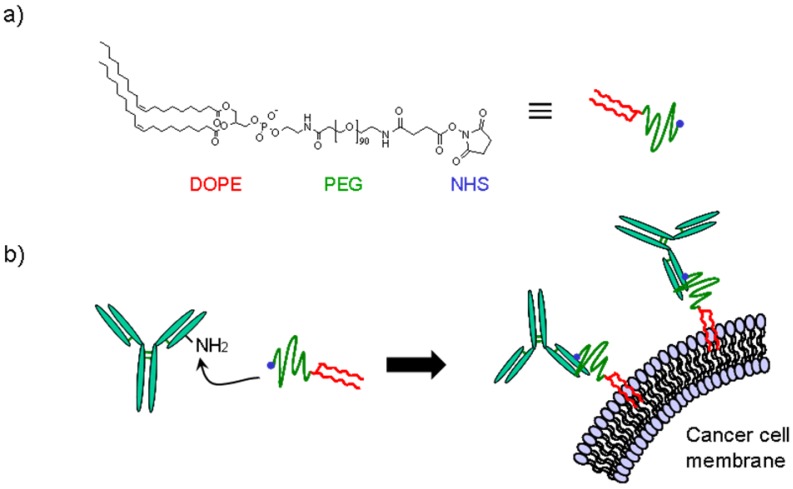
Chemical structure of BAM and a schematic diagram of the incorporation of BAM-conjugated IgG into the cell membrane.

In this study, to prepare an ATC-IgG complex using a simple and inexpensive approach, we used a poly(ethylene glycol)(PEG)-lipid for anchoring IgG onto the surface of ATCs (Figure 1). PEG-lipids consisting of a long PEG chain and a dioleylphosphatidylethanolamine (DOPE) were previously reported by us as a biocompatible anchor for biomembranes (abbreviated as BAM) (Figure 1a) [15]. BAM-modified molecules can bind to any type of cell because the oleyl moieties can be inserted into the ubiquitous lipid bilayer membranes in a noncovalent manner [15]. By attaching to BAM, various biomolecules such as biotin [15], an antagonistic peptide [16] and green fluorescent protein [15] were spontaneously displayed on living cells without loss of their activities. Other groups have also modified cell surfaces with functional biomolecules using PEG-lipids [17,18,19]. From these reports, we hypothesized that arbitrary IgGs can be readily displayed on ATCs by conjugating with BAM, and that phagocytosis of ATCs into DCs can be enhanced through the interaction between the IgG displayed on the surface of the ATCs and the FcγR expressed on DCs (Figure 1b). Initially, we optimized the conditions for IgG-BAM conjugation. The IgG-BAM conjugates prepared under various conditions were tested for their incorporation into cancer cells, and the incorporation rate was investigated using flow cytometry. We then confirmed that IgG-BAM treatment could enhance phagocytosis of ATCs by co-culturing with DCs at the same level as tumor-specific antibody treatment.

## 2. Experimental Section

### 2.1. Chemicals, Antibodies and Cell-Lines

All chemicals were commercially available and used as supplied without further purification. Purified mouse IgG_1_ was purchased from Beckman Coulter (Fullerton, CA, USA). Anti-human tumor cell specific antibody (SF-25) was purified from hybridoma-induced mouse ascites by protein A-Sepharose chromatography. For immunostaining of DC, phycoerythrin (PE)-conjugated mouse anti-CD11c antibody (eBioscience, San Diego, CA, USA) was used. Cy3 or Cy5-labeled anti-mouse Fc antibody (Jackson ImmunoResearch laboratories, West Grove, PA, USA) was used for staining Fc. Cell lines, HeLa and Ba/F3 were purchased from ATCC (Manassas, VA, USA). HepG2, B16 and the hybridoma secreting SF-25 were kindly provided by Tella, Inc (Tokyo, Japan). These cells were cultured in RPMI1640 or DMEM (Nissui, Tokyo, Japan) supplemented with 10% FBS, 2.05 mM L-glutamine, 30 μg/mL kanamycin and 0.2% NaHCO_3_.

### 2.2. Synthesis of BAM

BAM was synthesized by coupling dioleylphosphatidylethanolamine (DOPE, COATSOME ME-8181 from NOF Corporation, Tokyo, Japan) and PEG disuccinimidylglutarate (SUNBRIGHT DE-050GS from NOF Corporation). DOPE (17.6 mg, 28.8 μmol) and PEG disuccinimidylglutarate (151 mg, 30.2 μmol) were dissolved in 10 mL of anhydrous dichloromethane. Distilled triethylamine (60 μL, 431 μmol) was added, and the mixture was left to stir at room temperature for 1 h. After checking the consumption of DOPE on a thin layer chromatography, the crude product was precipitated by adding into 200 mL of diethyl ether, followed with centrifugation. The supernatant was removed by decantation. Vacuum drying the precipitation yielded white solid (132 mg, 84%).

### 2.3. Preparation of BAM-Conjugated IgG

BAM-conjugated IgG (IgG-BAM) was prepared by reacting mouse IgG_1 _and BAM in borate buffer (100 mM, pH 8.3) including 2.5% DMSO. The final concentration of IgG_1_ was 1 mg/mL (6.67 μM), and that of BAM was varied from 6.60 to 330 μM. The reaction mixture was incubated for 1 h at room temperature and the reaction was quenched by adding Tris-HCl buffer (pH 8.0) to give a final concentration of 50 mM. The product solution was purified by dialysis against PBS to remove unconjugated BAM, DMSO and a by-product. The purified products were analyzed by sodium dodecyl sulfate-polyacrylamide gel electrophoresis (SDS-PAGE) and matrix-assisted laser desorption/ionization-time-of-flight mass spectrometry (MALDI-TOF MS) with Autoflex Speed (Bruker Daltonics, Leipzig, Germany).

### 2.4. Incorporation of IgG-BAM Conjugates onto Cell Membrane

The cancer cells (1 × 10^6^ cells) were incubated in 100 μL of IgG-BAM solutions (1 μM in PBS) at room temperature for 15 min. As control experiments, cells were treated with IgG, BAM, or SF-25 solutions under the same condition, respectively. After treatment, the cells were washed twice with PBS. To evaluate the incorporation of IgG into the cell membrane, the IgG on the cell surfaces were fluorescently stained by Cy3-labeled anti-mouse Fc antibody according to standard protocols, and red-fluorescent cells were analyzed as Fc-positive cells by flow cytometry with a flow cytometer FACS Calibur using the CellQuest software (BD Labware, Franklin Lakes, NJ, USA). Additionally, the stained cells were observed with a confocal laser scanning microscope (CLSM) (TCS-NT, Leica Microsystems, Bensheim, Germany).

### 2.5. Generation of Bone Marrow-derived DCs

Bone marrow cells were obtained from female C57BL/6 mice (10-week old, from Charles River Breeding Laboratories, Tokyo, Japan) in accordance with a protocol approved by the Animal Care and Use Committee of the University of Tokyo. After red cells were lysed with ACK lysing buffer (Lonza, Fair Lawn, NJ, USA), bone marrow cells were cultured at 1 × 10^6^ cells/mL in RPMI medium (HyClone, Logan, UT, USA) in the presence of 20 ng/mL murine recombinant GM-CSF (rGM-CSF) (Peprotech, London, UK). The cultures were fed by replacing 75% of the medium with a fresh rGM-CSF-containing medium on days 1 and 4. Non-adherent cells and weakly adherent cells on day 6 of culture were harvested by strong pipetting and used as DCs in the following experiments.

### 2.6. Phagocytosis of Apoptotic Cancer Cells by DCs

HeLa cells were fluorescently stained with 5- or 6-(*N*-Succinimidyloxycarbonyl)-fluorescein 3',6'-diacetate (CFSE; Dojindo, Kumamoto, Japan) for 5 min. These stained HeLa cells were irradiated with ultra-violet (UV) light for 5 min and incubated for 6 h to induce apoptosis. Then, to incorporate IgG into the cell membrane, apoptotic HeLa cells were treated with IgG-BAM as described above. As control experiments, apoptotic HeLa cells were also treated with BAM and SF-25. After treatment, apoptotic HeLa cells (2 × 10^5^ cells) were co-cultured with DCs (1 × 10^6^ cells) in 500 μL of RPMI medium. After co-culturing for 3 h, all cells were treated with a PE-conjugated anti-CD11c antibody for fluorescently-staining DCs. The cells were observed with CLSM and quantitatively analyzed using FACS, as described above. The cells possessing both green and red fluorescence were identified as the DC phagocytizing apoptotic HeLa cells [20]. The phagocytosis rate of the DCs was determined by calculating the ratio of the double fluorescent-positive cells to the total red fluorescent-positive cells.

## 3. Results and Discussion

### 3.1. Conjugation of IgG and BAM

**Figure 2:**
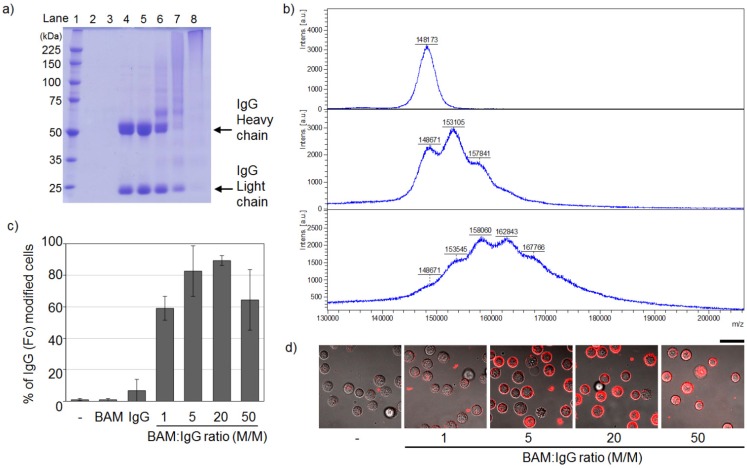
Optimization of the condition of IgG-BAM conjugation by changing the final molar ratio of BAM to IgG. (**a**) Qualitative analysis of the attachment of BAM to IgG by a reducing 7.5% polyacrylamide SDS-PAGE gel stained with Coomassie brilliant blue. Lane 1, protein molecular weight markers in kDa; lane 2, PBS as a control; lane 3, BAM as a control; lane 4, IgG as a control; lanes 5−8; IgG treated with BAM at various ratios: 1:1, 1:5, 1:20 and 1:50; (**b**) MALDI-TOF MS spectra of intact IgG (top), IgG treated with BAM at the IgG:BAM ratio of 1:5 (middle) and 1:20 (bottom); (**c**) Qualitative analysis of the incorporation of IgG-BAM conjugates into the membrane of HeLa cells by flow cytometry. (n = 3); (**d**) CLSM observation. Bar = 40 mm.

The process of conjugating IgG and BAM was optimized by changing the final molar ratio of BAM to IgG. The BAM molecule has an amine-reactive group at the end of the PEG chain and opposite to the lipid moiety, and reacts with lysine residues on IgG (Figure 1). Such random amine-coupling reactions onto IgG had been extensively used as a standard modification method because of their simplicity [21,22]. In the context of medical applications of PEGylated IgGs, the effects of random PEGylation on the effector function of IgG have been studied. Many studies using IgG modified with PEG of several kDa showed that FcγR binding to IgG was inhibited at high conjugation ratios of PEGs per IgG [21]. Therefore, to minimize the inhibition of FcγR binding by attachment of BAM, the condition of IgG-BAM conjugation was optimized by searching for a suitable conjugation ratio of BAM per IgG. The degree of attachment of BAM to IgG was qualitatively analyzed by SDS-PAGE (Figure 2a). As the molar ratio of BAM to IgG increased, the two bands derived from the heavy and light chains of IgG shifted to higher-molecular-weight species and broadened (Figure 2a, lanes 5−7), and finally, at the IgG:BAM ratio of 1:50, the bands of IgG disappeared and barely migrated onto the SDS-PAGE (Figure 2a, lane 8). In a previous report, a similar shift and broadening was observed in the band patterns of IgG modified with 5-kDa PEGs, and these changes were shown to correspond to the degree of PEG attachment [23]. Furthermore, MALDI-TOF MS analysis showed that IgG-BAM conjugation at the IgG:BAM ratio of 1:5 yielded IgGs with zero, one and two PEGs per IgG (Figure 2b, middle). At that of 1:20, a broaden peak was observed around the molecular weight of IgG with from one to five PEGs per IgG in the MS spectrum (Figure 2b, bottom). Thus, the present result indicates that IgGs bearing various amounts of BAM were successfully prepared by changing the molar ratio of BAM to IgG from 1 to 50.

### 3.2. Incorporation of IgG-BAM Conjugates onto the Cell Membrane

Incorporation of IgG-BAM conjugates onto the membrane of cancer cells was confirmed by flow cytometer analysis. HeLa cells treated with IgG-BAM conjugates were stained with a fluorescent-labeled anti-mouse Fc antibody, and then, the ratio of Fc-positive cells was determined by flow cytometry. Compared with the control cells (treated with only IgG), the Fc-positive ratios of the cells treated with IgG-BAM conjugates effectively increased (Figure 2c). In particular, treatment with the IgG-BAM conjugated at the IgG:BAM ratio of 1:20 gave the largest Fc-positive ratio, up to ~90%. Similarly, CLSM observations clearly indicated that IgG-BAM conjugates at high conjugation ratios of BAM could effectively incorporate into the cell membrane (Figure 2d).

On the other hand, although the IgG-BAM conjugated at the IgG:BAM ratio of 1:50 should bear the largest amount of BAM, the Fc-positive ratio was inclined to be smaller than that conjugated at the IgG: BAM ratio of 1:20 (*p* < 0.08). This is probably because the binding of fluorescent-labeled anti-Fc antibody to IgG was inhibited with the conjugated BAM at high conjugation ratios of BAM per IgG. As described above, in the case of random PEGylation of IgG, similar inhibition was found to be dependent on the conjugation ratio [21]. In addition, when the BAM modified near the Fc domain of antibody was inserted into the cell membrane, the fluorescent-labeled anti-Fc antibody would be unable to approach the Fc domain because the Fc domain was buried in cell membrane. The use of high conjugation ratios of BAM per IgG may increase the chance of modification near the Fc domain with BAM, thereby blocking the interaction with the anti-Fc antibody. From these results, we performed further experiments using the IgG-BAM conjugate prepared at the IgG:BAM ratio of 1:20.

**Figure 3:**
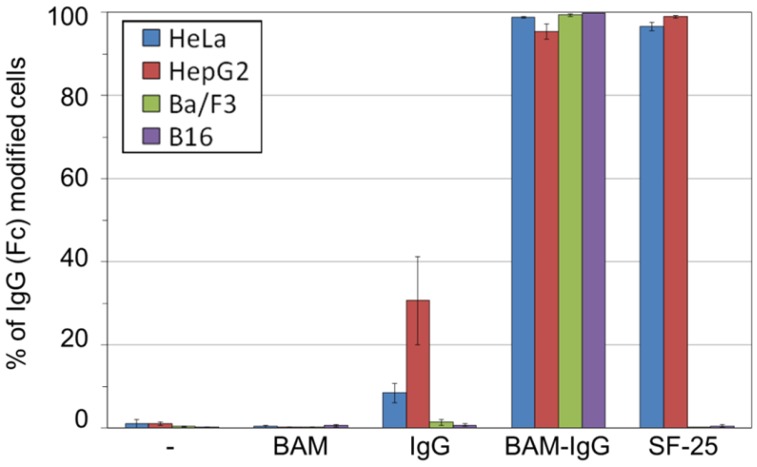
Fc-positive ratios of various cell lines analyzed by flow cytometry.

To demonstrate the versatility of the present method, a large variety of cell lines including HepG2, Ba/F3 and B16 were treated with the IgG-BAM conjugate. We selected HepG2 as a second human adenocarcinoma, Ba/F3 as a non-adherent cell and B16 as a murine cancer cell. Figure 3 shows that the Fc-positive ratios of all treated cell lines were almost 100%, whereas those of the cells treated with intact IgG were <30%. From this result, the IgG-BAM conjugate was successfully incorporated into the cell membrane through the interaction between BAM and the cell membrane, regardless of the cellular characteristics such as cellular derivation and adhesion properties. This is because the dioleyl moiety of BAM interacts with the ubiquitous lipid bilayer of cells.

The efficiency of the presentation of Fc on the cell surface was almost the same between IgG-BAM and the tumor-specific monoclonal antibody SF-25 (Figure 3). SF-25 was reported to bind a wide range of human cancer cell lines [24]. As such, the use of specific IgG against a ubiquitous antigen on cancer cells may be applied in the preparation of ATC-IgG complexes and be effective against a wide range of tumors. However, such antigen-specific monoclonal IgGs have limited medical applications. In particular, all antigen-specific monoclonal IgGs are obtained from animals including mouse, rabbit and goat. Therefore, the humanization of the IgG is required to avoid immune activation in patients and for enhancing effector cell activation [25]. In contrast, using the present BAM-conjugation method, arbitrary human IgGs obtained from blood can be employed. Thus, IgG-BAM is a promising tool for supplying safe ATC-IgG complexes at a low cost.

### 3.3. Effects of Incorporated IgG-BAM on Phagocytosis by Dendritic Cells

**Figure 4:**
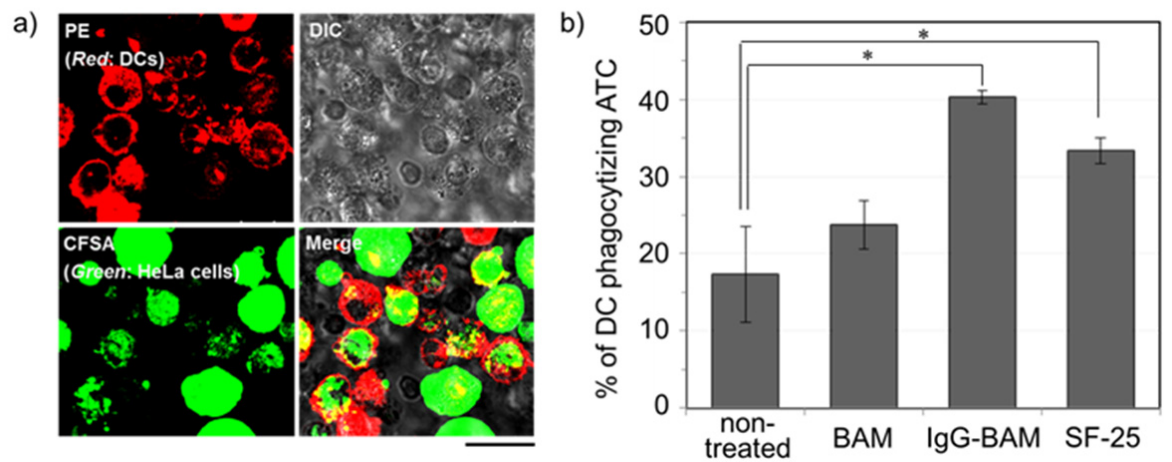
Analysis of the effects of the incorporated IgG-BAM on phagocytosis by DCs; (**a**) CLSM image of the DCs co-cultured with the ATCs treated with IgG-BAM. Bar = 20 mm; (**b**) The ratio of DC phagocytizing ATC analyzed by flow cytometry. (n = 3, * *p* < 0.05.)

To evaluate whether treatment of IgG-BAM can enhance the uptake of ATCs by DC, the rate of phagocytosis was analyzed after co-culturing the treated ATCs with immature DCs. The ATCs and DCs were stained with a green fluorogenic reagent, CFSE, and a red fluorescent protein-conjugated anti-CD11c antibody, respectively. After co-culturing, the cells possessing both green and red fluorescence were identified as the DCs that had phagocytized ATCs [20], and such double-positive cells were observed with CLSM and quantitatively analyzed using flow cytometry (Figure 4). Figure 4a shows the CLSM image of the DCs cocultured with the ATCs treated with IgG-BAM. In the red-fluorescent image, the outer membrane of the DCs was stained red due to the attachment of the PE-conjugated anti-CD11c antibody. In contrast, in the green image, the whole ATCs fluoresced green. In the merged image, several red-stained cells were observed to have incorporated green-stained bodies. Consequently, the florescent images clearly confirm that DCs have phagocytized ATCs.

Figure 4b shows the phagocytosis rate of DCs under various conditions of treatment for ATCs. Compared with no treatment, treatment with IgG-BAM increased the phagocytosis rate more than two-fold. For over forty years, PEG has been commonly used as an effective fusogenic reagent for somatic cells [26]. As a control experiment, we have investigated the effect of treatment with only BAM on the uptake of ATCs. Treatment with only BAM slightly increased the phagocytosis rate (Figure 4b). These results strongly suggest that the enhancement effect of IgG-BAM is not derived from the fusogenic properties of the PEG moiety but from the effective presentation of IgG on the ATC surface. In a previous report on ATC chemically-modified IgG, the phagocytosis rate was enhanced almost two-fold by IgG modification [7]. Thus, the present enhancement of DC phagocytosis by IgG-BAM treatment is essentially the same as that observed when IgG is chemical modified. However, in contrast to chemical modification approaches, the present IgG-BAM treatment has the distinct advantage of enhancing phagocytosis without altering any chemical structures on the cell-surface antigens. There are many well-known cell surface antigens present on tumor cells, such as MUC-1 and CCA-1. Alteration in the chemical properties of such cell surface antigens may lead to decreases in the repertoire of antigenic epitopes for presentation to anti-tumor effector and mediator cells. On the other hand, from previous reports about vaccination with PEGylated liposomes encapsulating antigens, intracellular processing of antigen remained virtually unaffected by the presence of PEG-lipids [27,28,29]. Thus, noncovalent treatment with IgG-BAM is a promising method for activating a broad repertoire of anti-tumor immunocytes.

As shown in Figure 4, the enhancement effect when using IgG-BAM was almost the same as observed for SF-25. Since IgG-BAM incorporates anywhere on the surface of the lipid bilayer of ATCs, the amount of presented IgG on ATCs is presumably limited only by the surface area of the ATC, whereas the binding amount of Ag-specific IgG, such as SF-25, is limited by the surface amount of Ag. Thus, treatment with IgG-BAM has the potential advantage of presenting a relatively large amount of IgG on the cell surface. Conversely, IgG-BAM potentially has a disadvantage in its immunological effector activities. As described above, random PEGylation of IgG has been reported to decrease the effector activities of IgG through interfering with FcγR binding [21]. Presumably, random BAM modification similarly interferes in the interaction between the Fc domain on ATCs and the FcγR on DCs. In the present study, treatment with IgG-BAM may exert an enhancing effect that was comparable with SF-25. This is probably because optimization of the BAM-conjugation condition minimized the disadvantage of IgG-BAM in the mean affinity with FcγR, and the advantage in the presentation amount countered this potential disadvantage.

## 4. Conclusions

In the present study, arbitrary IgG was presented on cancer cell surfaces by conjugating with a PEG-lipid, BAM. Regardless of the cellular characteristics, the present method using the IgG-BAM conjugate was successfully applied to all four kinds of mammalian cells. Furthermore, ATCs treated with IgG-BAM were more efficiently phagocytosed by DCs than untreated ATCs *ex vivo*. This enhancement rate of treatment with IgG-BAM was essentially identical to the treatment rate with an Ag-specific IgG. This result indicates that IgG-BAM treatment enhances the efficacy of ATC-based DC vaccinations without the requirement of an expensive Ag-specific IgG. In addition, because the present BAM-conjugation method is potentially applicable to human arbitrary IgG, IgG-BAM treatment represents a promising method for generating more effective DC vaccine therapies in clinical trials.
